# Gastrointestinal Bleeding in Patients With Acute Ischemic Stroke: A Literature Review

**DOI:** 10.7759/cureus.53210

**Published:** 2024-01-30

**Authors:** Muhammad Ali Aziz, Srikaran Bojja, Ahmed Ali Aziz, Nismat Javed, Harish Patel

**Affiliations:** 1 Internal Medicine, BronxCare Health System, New York City, USA; 2 Internal Medicine, BronxCare Health System, Icahn School of Medicine at Mount Sinai, New York City, USA; 3 Internal Medicine, Capital Health Regional Medical Center, Trenton, USA; 4 Gastroenterology and Hepatology, BronxCare Health System, New York City, USA

**Keywords:** gastrointestinal hemorrhage, ischemic stroke, cerebrovascular accident, gastrointestinal bleeding, anticoagulants

## Abstract

Stroke is an infarction of the central nervous system (brain, spinal cord, or retina) that results from a disruption in cerebral blood flow either due to ischemia or hemorrhage. Complications of acute stroke are common and include pneumonia, urinary tract infection, myocardial infarction, deep vein thrombosis, and pulmonary embolism, among several others, all of which increase the risk of poor clinical outcomes. Gastrointestinal bleeding is a well-known complication that can occur during the acute phase of stroke. In this review, we have summarized the existing data regarding the incidence, pathophysiology, risk factors, morbidity, mortality, and management strategies for gastrointestinal bleeding in patients with acute ischemic stroke.

## Introduction and background

Stroke is an infarction of the central nervous system (CNS; brain, spinal cord, or retina) that results from a disruption in cerebral blood flow either due to ischemia or hemorrhage with symptoms persisting for ≥24 hours. The diagnosis can be made by clinical symptoms, imaging, or pathological evidence [1]. Stroke can be classified by the age of the affected individual, the location involved, the vascular territory affected, and the etiology of the stroke. Etiology is the most common way of classifying stroke. Ischemic stroke occurs due to obstruction of blood vessels by thrombosis, embolism, or systemic hypoperfusion. The American Heart Association (AHA) defines ischemic stroke as “an episode of neurological dysfunction caused by focal cerebral, spinal, or retinal infarction” [1]. Around 87% of all strokes are ischemic strokes [2]. Hemorrhagic stroke occurs due to the rupture of blood vessels and leakage of blood into the CNS parenchyma, leading to cell death. Hemorrhagic stroke can either be due to intracerebral hemorrhage or subarachnoid hemorrhage. The incidence of stroke is more than 795,000 per year in the United States, and around 140,000 Americans die each year as a result of stroke [2]. Ischemic stroke is associated with several complications including pneumonia, urinary tract infection (UTI), myocardial infarction, deep vein thrombosis (DVT), and pulmonary embolism (PE), among several others [3]. Gastrointestinal bleeding (GIB) is a well-documented complication of stroke and is associated with an increased risk of morbidity and both short-term and long-term mortality [4-7].

The objectives of this review are to discuss the incidence, pathophysiology, risk factors, morbidity, mortality, and management strategies for GIB in patients with acute ischemic stroke (AIS). We conducted a scoping review of the literature from PubMed and Google Scholar using the keywords “gastrointestinal bleeding,” “acute ischemic stroke,” “treatment,” and “prognosis.”

## Review

Incidence

The incidence of GIB in patients with AIS has been fairly variable across different studies. For instance, in a retrospective study of 6,853 patients with AIS in Canada, O’Donnell et al. observed that 1.5% of the patients experienced GIB during hospitalization [6]. Hsu et al., on the other hand, worked with an Asian cohort and found the incidence of GIB in patients with AIS close to 7.8% [5]. Similarly, Rumalla and Mittal [8] found that patients of Asian descent, after adjusting for confounders, were more likely to experience GIB following an AIS. This observation could be due to the increased prevalence of *Helicobacter pylori* infection in Asian populations [5]. It is interesting to note that according to Chen et al. [9], gastroduodenal ulcers develop in 44% of all patients admitted to neurological intensive care units with a diagnosis of AIS.

Onset

GIB usually occurs within one week after stroke onset [10]. The source of bleeding in AIS includes both the upper and the lower GI tract. Ogata et al. worked with a Japanese cohort that suffered from AIS and found that 50.4% of the cases had an upper GI source, including peptic ulceration, malignancies, reflux esophagitis, Mallory-Weiss syndrome and esophageal varices, while 25.8% of the patients were identified with a lower GI tract source including pseudomembranous colitis, ischemic colitis, angiodysplasia, polyps, and diverticula. Also, 23.6% had an unknown source of bleeding after colonoscopy and endoscopy [10].

Pathophysiological of GIB in AIS

Several pathophysiological mechanisms have been proposed to explain the association of GIB with acute cerebral infarction. Prior studies indicated that stroke leads to vagal hyperactivity, which would result in increased gastric acid and pepsin secretion and damage of gastrointestinal (GI) mucosa, leading to stress ulcerogenesis [5,11]. A review by Camara-Lemarroy et al [3] found that stroke leads to systemic inflammation, oxidative stress, inhibition of the nitric oxide pathway, and use of antiplatelet drugs, all of which can contribute to ulcerogenesis and result in post-stroke GIB. Similarly, Fu [12] stated that stroke results in the activation of noradrenaline neurons and a dysregulation of the CNS’s supply to the digestive system. This has been proposed to increase the risk of mucosal injury in the digestive system. This could also explain why a severe ischemic stroke is associated with an increased risk of GIB. We know that the autonomic nervous system pathway descends from the hypothalamus to the spinal cord via the midbrain. This can explain the association between posterior circulation ischemia and GIB [13]. Schaller et al. suggested that there is a catecholamine surge following AIS. The vasoconstriction may lead to mucosal ischemia, and eventually to GIB [14]. Izumiyama and Kogure found that during an ischemic stroke, there is a reduction in gastric mucosal blood flow, which can contribute to ulcerogenesis [15].

Risk factors

Over the years, several studies have been conducted to help characterize the risk factors for GIB in patients with AIS. The identification of risk factors can help clinicians risk-stratify their patients to enable the allocation of medical resources to patients who are at a higher risk for GIB (Table 1).

**Table 1:** Factors associated with an increased risk of gastrointestinal bleeding in acute ischemic stroke GCS, Glasgow Coma Scale; NIHSS, National Institute of Health Stroke Scale; NSAID, Nonsteroidal anti-inflammatory drug; TACI, Total anterior circulation infarct; UTI, urinary tract infection; WBC, white blood cell

Factors
Older age
Male
Impaired activities of daily living, pre-stroke dependence
History of peptic ulcer disease
High baseline NIHSS score
Severity of the stroke
Baseline neurological deficit
Low GCS score, altered mental status
Concomitant infection (pneumonia, UTI)
Sepsis
Posterior circulation infarction
Middle cerebral artery ischemia
TACI
Elevated WBC count
Carotid artery stenosis
Elevated fasting blood glucose levels
History of malignancy
Coagulopathy, atrial fibrillation
Cardioembolic stroke
Renal insufficiency
Hepatic insufficiency
Fluid and electrolyte disorders
Paralysis
Alcohol abuse
Iron deficiency anemia
NSAIDs and aspirin use
Antiplatelet use
History of hypertension
History of gastrointestinal bleeding
Preexisting coronary heart disease
Prior history of stroke
Family history of stroke

Patients with ischemic stroke who experienced GIB were older [4,6,10,12,13,16,17], more likely to be male [13], and more likely to have impaired activities of daily living before onset [10]. A history of peptic ulcer disease was also found to be associated with GIB in patients with AIS. It is important to note that GIB developed in those with prior peptic ulceration despite acid-suppressing therapy [10]. Additionally, the National Institutes of Health Stroke Scale (NIHSS) score on admission was significantly higher in those with GIB, suggesting that the severity of the stroke and baseline neurological deficit was associated with an increased risk of GIB [10,12]. A history of hypertension and GIB were also associated with an increased risk [13].

Fu [12] found additional independent risk factors for GIB in patients with acute cerebral infarction, including a low Glasgow Coma Scale (GCS) score, infection, and posterior circulation infarction [18]. Pneumonia and UTIs were the most frequent infections encountered in patients with GIB [19]. An elevated white blood cell (WBC) count, an increased prevalence of carotid artery stenosis, and elevated fasting blood glucose levels were also documented among stroke patients with GIB compared to stroke patients without GIB [12]. Middle cerebral artery ischemia and total anterior circulation infarct were associated with a higher incidence of GIB, but partial anterior circulation infarct was not [13]. A history of malignancy and coagulopathy are also significant risk factors for AIS-related GIB [6,10,13]. The prevalence of atrial fibrillation was significantly higher in patients with GIB compared with those without GIB [5,6,8,10,12,16,20]. Other factors such as cardioembolic stroke, preexisting coronary heart disease, prior history of stroke, and family history of stroke were also more prevalent in patients with GIB compared with those without GIB [10,19].

One explanation why atrial fibrillation, carotid artery stenosis, and cardioembolic stroke are more prevalent in patients with GIB is that these factors are often associated with an increased severity of stroke, and severe strokes increase the risk of GIB [21,22]. Similarly, an altered mental status (indicated by a low GCS score) indicates a severe stroke as well [5,12].

Infection, sepsis, and an elevated WBC count have all been associated with an increased risk of GIB in AIS [12]. One explanation could be that sepsis causes splanchnic hypo-perfusion and mucosal ulceration due to pro-inflammatory cytokine release [23,24].

Renal insufficiency and hepatic insufficiency are also important risk factors for both upper and lower GIB in patients with AIS [16,25]. Hepatic cirrhosis causes esophagogastric varices secondary to portal hypertension and also causes coagulopathy secondary to hepatic dysfunction, both of which can contribute to GIB following AIS [13]. Similarly, renal insufficiency leads to platelet dysfunction, which can lead to a bleeding tendency [26].

Rumalla and Mittal used a larger cohort to help identify risk factors for poststroke GIB that were not previously described [8]. They found that people with fluid and electrolyte disorders, paralysis, alcohol abuse, and iron deficiency anemia were more likely to develop GIB following AIS.

Long-term use of nonsteroidal anti-inflammatory drugs (NSAIDs) and aspirin is also considered to be associated with GIB after AIS in a study by Wijdicks et al. [7].

Although steroid use before stroke onset was more frequent in patients with GIB, the association was statistically marginal after adjustment for confounders [10]. However, patients who were treated with statins prior to stroke did have a statistically significant decreased risk of developing GIB [10].

Dyslipidemia was found to be associated with a reduced risk of GIB [10]. One explanation could be that the eradication of *H. pylori* leads to elevated serum levels of cholesterol or triglyceride and hence dyslipidemia [27-29], and peptic ulcer disease due to infection with *H. pylori* is associated with an increased risk of GIB in AIS [10].

Hsu et al. showed in their Asian cohort that patients with multiple risk factors had a higher incidence of hemorrhage [5]. This suggests that the frequency of hemorrhage may be directly associated with the number of risk factors. This idea was carried forward in 2014 by Ji et al. who developed an 18-point AIS-GIB score by utilizing the China National Stroke Registry using independent predictors of GIB, including age, gender, history of hypertension, hepatic cirrhosis, peptic ulcer, history of GIB, pre-stroke dependence, NIHSS score on admission, GCS score on admission, and stroke subtype. This score can help predict the expected in-hospital GIB at presentation and help identify vulnerable patients. The higher the score, the more vulnerable the patient to develop GIB [13].

Morbidity and mortality

GIB in AIS is associated with increased morbidity and mortality and a worse prognosis. According to Ogata et al. and Fu et al., GIB was independently associated with neurological deterioration, in-hospital death, increased length of hospital stay, poor functional outcome, increased disability level at three months, increased risk of one-year mortality, and an increased risk of all-cause mortality during hospitalization [10,12]. One proposed mechanism why GIB is associated with progressive neurological deterioration is that bleeding is managed by withdrawing of antithrombotic therapy that leads to a prothrombotic state [30]. A prothrombotic state may result in recurrent strokes, further decline in neurological symptoms, and poor clinical outcomes.

O’Donnell et al. found that GIB was associated with a threefold increase in the odds of death or severe dependence at discharge and a 1.5-fold increase in mortality at six months. Importantly, GIB remained a strong predictor of death or dependency at discharge and mortality at six months, even when adjusted for baseline confounders and in-hospital medical complications that included recurrent stroke, myocardial infarction, venous thromboembolism, pneumonia, and UTI [6]. In fact, GIB was found to be an independent risk factor of death within one year after stroke in addition to infection, modified Rankin Scale grade ≥ 4, and coronary heart disease [12].

Chou et al. studied the long-term mortality rates in patients with GIB in AIS. He demonstrated that GIB in AIS was associated with an increased three-year mortality in patients with first-ever ischemic stroke. This association suggests that ischemic stroke patients who developed GIB did poorly in the long run and had a higher long-term mortality rate [16].

Du et al. found that GIB was associated with an approximately 1.5-fold higher risk of stroke recurrence after AIS at three months, six months, and 12 months [19]. This may be an indirect consequence of decreased antithrombotic therapy use in patients with GIB, although that is unlikely because the association still persisted after adjusting for the use of antithrombotic therapy. The association also existed after adjusting for other confounders and co-morbidities such as age, gender, NIHSS on admission, hypertension, hyperlipidemia, diabetes mellitus, coronary artery disease, history of transient ischemic attack (TIA) or stroke, family history of stroke, atrial fibrillation, and prior history of smoking and heavy alcohol abuse.

Several studies have noted a bidirectional association between GIB and a myriad of in-patient complications including pneumonia, DVT, PE, UTI, septicemia, and acute kidney injury [6,31]. For instance, the treatment of GIB requires the cessation of anticoagulant medications, which leads to a prothrombotic state and an increased propensity to develop DVTs and PE. The treatment of DVT and PE requires the use of anticoagulants, which can predispose patients to developing GIB [8]. Liebler et al. described an increased risk of developing pneumonia in critically ill patients with upper GIB. He also demonstrated a poor outcome for pneumonia patients with an upper GIB [32]. Stress ulcer prophylaxis is effective in reducing the risk of bleeding, and its use is associated with a lower rate of nosocomial pneumonia and mortality [33].

Chou et al. showed that the hemoglobin level was significantly lower in the group that presented with GIB when compared to those without GIB on admission [16]. One explanation could be that small amount of bleeding may occur chronically before the onset of stroke in patients with GIB [34]. Ogata et al. went further to show that during their hospital stay, the hemoglobin concentration of patients with GIB fell further by a median value of 2.5 g/dL and around 31.5% of the patients with GIB had to undergo blood transfusion [10].

Rumalla and Mittal observed that patients with GIB were more likely to undergo invasive procedures such as intubation and mechanical ventilation, tracheostomy, gastrostomy, and blood transfusion [8].

Several mechanisms have been proposed to explain the increased morbidity and mortality observed with major bleeding. First, the hemodynamic effect of major bleeding is likely to be an important determinant of brain ischemia [34]. Second, withdrawal of antithrombotic therapy, when bleeding occurs, is likely to increase the risk of myocardial infarction, venous thromboembolism, and ischemic stroke [35]. Third, bleeding may also induce a prothrombotic state by causing platelet and coagulation activation, which would be expected to increase the risk of thrombotic events [35]. Finally, blood transfusion has been associated with increased mortality in a number of studies, including a clinical trial [23,36-38].

Management

The prevention and treatment of post-stroke GIB is complicated because it contradicts the prevention of recurrent stroke where commonly antiplatelet or anticoagulation agents are used. One strategy as proposed by Siddiqui et al. is to risk-stratify patients with GIB via endoscopic evaluation [39]. Although acute AIS is considered a relative contraindication to GI endoscopy (GIE), Siddiqui et al. used the Nationwide Inpatient Sample (NIS) database - the largest national inpatient database in the USA - to show that patients with AIS and concurrent GIB who undergo evaluation via endoscopy have lower in-hospital mortality, a shorter in-hospital length of stay, and consequently lower total hospitalization cost and reduced utilization of health care resources when compared to those who do not undergo GIE even after stratifying for potential confounders. A possible explanation for these findings is that GIE can help localize and, if required, control the source of the GIB and help reduce morbidity and mortality associated with bleeding. Additionally, GIE allows physicians to risk-stratify patients with AIS to receive antithrombotic therapy. GIE can help localize the source of bleeding, determine the severity of the lesion and the bleeding, help evaluate the risk of rebleeding, and guide the use of antithrombotic therapy. Patients who are less likely to develop a rebleed can therefore be started on antiplatelet therapy helping the prevention of recurrent strokes [39].

Hsu et al. found the upper GI tract to be the major source of bleeding in their Asian cohort. In more than half of these patients, endoscopy revealed bleeding from epithelial erosions and ulcers - lesions similar to stress ulcers. These lesions are superficial and usually do not perforate thus suggesting that medical treatment alone might be adequate for GIB after stroke in select cases [5,31].

The role of aspirin and other NSAIDs in causing GIB in AIS is debatable due to the conflicting information available across various studies. It is interesting to note that although prior studies have found that the use of antithrombotic agents or NSAIDs prior to the stroke is associated with GIB, Ogata et al. observed no such association in their study in 2014 [5,10]. He also stated that the use of other antiplatelet agents was not associated with an increased risk of GIB.

In 2019, Fu reported similar results suggesting that the use of antiplatelet medications (aspirin and clopidogrel), anticoagulants (warfarin), and recombinant tissue plasminogen activator (rtPA) was not associated with an increased risk of GIB [12]. In addition, no significant correlation was found between prophylactic proton pump inhibitors (PPIs) and GIB, which contradicts prior studies where ulcerative prophylaxis was effective in reducing the incidence of GIB [40]. In fact, prior studies suggest that the prophylactic use of histamine-2 receptor blockers (H2RAs), PPIs, or sucralfate are all effective in reducing GIB in critically ill patients [41-43]. To confound the matter even further, Rumalla and Mittal observed that patients who received thrombolytic therapy for AIS were less likely to suffer from GIB than those who did not [8]. Similarly, after adjusting for confounders, Inohara et al. did not find an increased risk of mortality and bleeding in rtPA-treated AIS patients with recent GIB, suggesting that the use of rtPA is not necessarily a contraindication and can be considered in AIS patient with GIB [44].

As far as dual antiplatelet therapy goes, both aspirin-dipyridamole and aspirin-clopidogrel combinations are associated with an increased risk of GIB compared to monotherapy [45]. Specifically, Hilkens et al. showed that the initiation of dual antiplatelet therapy after a TIA or stroke is associated with a doubling of GIB risk in the first 30 days [46]. Interestingly though, this high early risk declines over time and is similar to aspirin one year after the stroke [47]. When comparing aspirin with other antiplatelet agents, cilostazol in particular has been shown to have fewer GIB events compared to aspirin, albeit a higher incidence of other GI adverse events [48,49].

Antithrombotic therapy is an integral part of prophylaxis against acute embolic events in patients with atrial fibrillation. When compared to warfarin, the ARISTOTLE trial demonstrated a lower overall risk of bleeding with apixaban, while the ROCKET AF trial and RE-LY trial showed a slightly higher risk of GIB with rivaroxaban and dabigatran [50-52].

Enteral nutrition has been studied in patients with AIS and GIB. According to previous studies, moderate feeding was noted to have a better prognosis in such a cohort of patients with patients having lower mortality rates and improved neurological function [53]. However, long-term enteral nutrition is a topic of debate. Patients on long-term enteral feeding through a percutaneous endoscopic gastrostomy tube were likely to demonstrate high mortality and poor outcomes as well as a poor quality of life [54].

## Conclusions

Stroke is a cause of major mortality and morbidity in the United States. Ischemic stroke can result in several complications, including pneumonia, UTIs, heart attacks, DVT, and PE. GIB, another complication, is associated with increased short- and long-term morbidity and mortality. Given the risk factors in this cohort, there is a high risk of concomitant GIB. The results from this literature review, along with earlier research, emphasize the need for future studies focusing on preventing GIB in AIS patients. These studies should explore screening methods and preventive treatments, offering guidance on the appropriate use of antiplatelet and antithrombotic therapies.
